# Linking Individual Patient Data to Estimate Incidence and Prevalence of Parkinson's Disease by Comparing Reports of Neurological Services and Pharmacy Prescription Refills at a Nationwide Level

**DOI:** 10.3389/fneur.2019.00640

**Published:** 2019-06-18

**Authors:** Szabolcs Szatmári, András Ajtay, Mónika Bálint, Annamária Takáts, Ferenc Oberfrank, Dániel Bereczki

**Affiliations:** ^1^János Szentágothai Doctoral School of Neurosciences, Semmelweis University, Budapest, Hungary; ^2^Department of Neurology, Semmelweis University, Budapest, Hungary; ^3^MTA-SE Neuroepidemiological Research Group, Budapest, Hungary; ^4^Hungarian Academy of Sciences Centre for Economic and Regional Studies, Budapest, Hungary; ^5^Institute of Experimental Medicine of the Hungarian Academy of Sciences, Budapest, Hungary

**Keywords:** Parkinson's disease, incidence, prevalence, record linkage, Hungary

## Abstract

**Objectives:** We set forth to estimate the number of those with Parkinson's disease (PD) in Hungary, a country with a single-payer health insurance system covering 10 million inhabitants.

**Methods:** We analyzed all hospital and outpatient reports from neurological services and pharmacy reports of prescription refills. We cross-checked clinically administered diagnosis of PD with prescription refills of antiparkinsonian medications using record linkage. We used the ICD-10 code of G20 in any diagnostic category to find all cases with possible PD. For case certification those patients were considered to have PD who were recorded with G20 code in at least 2 calendar years. For a more conservative estimation we determined the number of those who also refilled antiparkinsonian medication.

**Results:** Between 2010 and 2012 there were 46,383 subjects with certified PD by clinical criteria. Crude and age-standardized incidence were 49/100,000/year (95% CI: 45–53), and 56/100,000/year (95% CI: 51–60). Crude and age standardized prevalence rates were 404/100,000 (95% CI: 392–416) and 471/100,000 (95% CI: 456–485). Of all clinically certified PD patients 72% refilled antiparkinsonian medications.

**Discussion:** The incidence and prevalence of PD in Hungary is higher than earlier estimates, which should be considered in organizing healthcare services for this patient group.

## Introduction

Parkinson's disease (PD) is an increasing social and economic burden worldwide, reducing the quality of life of patients, being one of the leading causes of neurological disability in the adult population and being associated with large direct costs to society (1, 2).

Incidence and prevalence rates of PD vary greatly (3–5). Observed variations may result from different epidemiologic methods used, but might also be a consequence of environmental or genetic factors, differences in age, sex or ethnicity distributions of the study populations. Global estimates of PD prevalence is projected to increase dramatically in the upcoming years (6), therefore there is a growing need to continuously monitor the incidence and prevalence of the disease.

Generally there are two main designs for frequency estimation of PD: case finding studies, in which hospital, general practice or pharmacy records are searched for diagnosed cases, often in combination; and door-to-door surveys in which the studied population is screened for the presence of the disease using the same, standardized diagnostic criteria. The most reliable method is a door-to-door survey, however it requires greater effort and expense than case finding studies (7, 8).

Patients with particular medical conditions are often identified from healthcare administrative databases using the International Classification of Diseases (ICD-9, ICD-10) or pharmacy records. Such databases offer valuable information for health economic analyses, healthcare planning, and to a specific degree epidemiological investigations. Countries with a single-payer state health insurance system with full coverage of the total population have the possibility of epidemiological analyses at a nationwide level. In such analyses, the accuracy of case definitions needs to be taken into account (9).

Pharmacy data on the other hand are reliable sources of true drug exposure (10). As for PD, antiparkinsonian drugs (APDs) are reliable markers in pharmacy records to monitor the presence of the disease (11).

Thus, identifying a population of patients with PD with appropriate algorithms used from healthcare administrative databases and pharmacy data is a potentially practical, inexpensive strategy to develop large population based epidemiologic and health service studies (12, 13).

Hungary is a country with 10 million inhabitants and a single-payer state health insurance system covering the whole population. Since 1996 patients have been identified in this system by a 9-digit unique personal code number. This personal identifier is used in all healthcare services including hospital care, outpatient specialist care, general practice and pharmacy records.

Previous estimates three decades ago based on door-to-door survey in a district of the capital city estimated that around 12,300 people may live with PD in Hungary (14), and a European review reported 21 thousand people in Hungary with PD (15). However, epidemiological statistics of PD in the region are outdated and need revision.

In the present study, we calculated the incidence and prevalence rates of PD in Hungary and we cross checked the physician administered diagnosis of PD (ICD-10, code G20) by pharmacy refills of APDs among the total Hungarian population on an individual level using record linkage.

## Materials and Methods

In the framework of the Hungarian National Brain Research Program the NEUROHUN 2004–2017 database was created from medical and medication reports submitted for reimbursement purposes (16). In the present case PD was assessed using the database of the National Health Insurance Fund from neurology departments of all hospitals, neurology outpatient services and pharmacies throughout Hungary. The original patient identifier codes were anonymized and encrypted identifiers were used. The database from hospitals and outpatient services contains information covering a 10-years period between 2004 and 2013 while pharmacy data are available from 2010. All personal data protection regulations were followed, and the Ethics Committee of Semmelweis University, Budapest, Hungary approved the study (Approval No: SE TUKEB 88/2015).

There are two independent databases that we linked: the database of the neurological patient care system, and the pharmacy database of medication refills. Record linkage between the clinical and pharmacy databases was possible by applying the encrypted individual identifier.

The study was divided in four phases. Case identification in the first phase was followed by 3 steps of case certification. For case identification, all individuals were selected from the neurological care system covering 10 years (2004–2013), who used either the inpatient or the outpatient neurological service at least once during this period and had a primary or secondary diagnosis of PD (ICD-10, G20).

In the second phase, case certification was performed in the clinical database. The following criteria had to be fulfilled for a patient to be considered to have PD in the year of evaluation:

1. to receive G20 diagnosis in the neurology care system in at least 2 years during the 10-year period regardless of the diagnosis type (i.e., primary/admitting diagnosis or secondary diagnosis) and
2. to be alive at least 1 day in the year of evaluation.

We calculated crude and age-standardized incidence and prevalence rates of PD in Hungary for each year of the period 2010–2012. The rates were calculated on the data of the first diagnosis. As for the clinically certain PD diagnosis we required the appearance of the G20 code in the patient records at least in 2 years, we could not estimate incidence for the last year of the database i.e., for 2013.

In the third phase, validation of the clinical diagnosis criteria of PD on a smaller subsample was performed. Firstly, we made an IT validation of data by cross-checking the database of the national neurological patient care system (NEUROHUN) and the local integrated hospital healthcare IT system (MedSol) of Semmelweis University, Budapest and vice versa, by choosing a 2 months period for examination: November–December, 2012. First, we identified all patients at our department from the hospital records (MedSol) who were treated with an ICD-10 code of G20 either in the inpatient or the outpatient setting. Using the year of birth, the postal code of the residence, the gender, the admission and discharge dates we checked if these patients do or do not appear in the large database (NEUROHUN). Second, from the large database we identified those who appeared at least in 2 years of the study with G20 diagnosis, and one of these appearances occurred in the period of November-December 2012 at our department. We checked if these patients can be found in the local hospital records. Finally, in the same period we retrieved the individual patient healthcare documents of all inpatients (*N* = 31) and 19 consecutive outpatients with a clinical diagnosis of PD, checked if they appear at least in 2 years with G20 diagnosis code in the large database and we made sure that PD diagnosis is accurate and clinically supported based on final hospital discharge reports.

In the fourth phase, to achieve a more conservative case certification, we counted those who fulfill the clinical diagnosis criteria of PD and also refilled an APD prescription in the year of interest. APDs are coded N04 in accordance with the Anatomical Therapeutic Chemical Classification (ATC) system. N04A-anticholinergic agents as well as N04B-dopaminergic agents were included. Our analysis could evaluate prescriptions of N04 ATC drugs refilled at pharmacies, but had no access to inpatient medication use in hospitals. For this analysis first we identified all patients with any kind of diagnosis who refilled an APD between 2010 and 2012. Afterwards, among these patients, using record linkage we identified those individuals who had a PD according to our clinical criteria. This way we cross-checked and validated the clinically administered diagnosis of PD by pharmacy refills of APD of the same patient. The requirement of ADP refill in a certain year for case certification results in underestimating the total number of PD patients, by excluding those who still live but do not refill an APD in that certain year.

The rate of APD use in PD patients was calculated in two ways to find the minimum and maximum potential values:

(a) the minimal rate of APD use in PD patients was calculated as:

$$\begin{array}{l} \frac{\Sigma PD_{clin\;}{\cap \;\Sigma Refill}_{N04}}{\Sigma PD_{clinalive\;}} \end{array}$$

where the nominator is the number of PD patients where our clinical criteria is met and refilled at least once an N04 ATC medication, and the denominator is the number of patients with PD who are alive in the year given and meet our clinical criteria.

(b) the maximum rate of APD use in PD patients was calculated as:

$$\begin{array}{l} \frac{\Sigma PD_{clin\;}{\cap \;\Sigma Refill}_{N04}}{\Sigma PD_{clinalive\;\cap \;neurol\;}} \end{array}$$

where the nominator is the number of PD patients where our clinical criteria is met and refilled at least once an N04 ATC medication, and the denominator is the number of patients with PD who are not only alive but also presented at a neurology service in the given year.

Data extraction was performed by a research assistant IT specialist, with several years of experience in reviewing medical records of patients with neurological conditions. Results of individual searches in the database were exported to excel files, which were used for the custom query for the final analysis of PD patients. The research team was not blinded to the presence or absence of G20 or other ICD-10 codes either in the clinical or in the pharmacy data.

### Statistical Analysis

The crude incidence and prevalence rates were standardized to the European standard population of 2013 with 95% confidence intervals (CI 95%) using a binominal distribution (17). Calculating incidence and prevalence we considered those who have PD who fulfill our diagnostic criteria (received G20 diagnoses in at least 2 years).

## Results

### Estimating the Number of Possible PD Patients Over 10 Years

Out of the 10 million inhabitants of Hungary, 2.9 million people used at least once an inpatient or outpatient neurological service in the 10-year period of 2004–2013. During this time there were overall 96,874 patients admitted to hospitals or had outpatient visits at a neurological specialist care at least once with PD, ICD-10 code G20, as a primary or secondary diagnosis. Patients with parkinsonism (PKM, only G21–26 diagnoses without any appearance with G20) are not included in this number. Of the 96,874 patients with ICD-10 code G20, as a primary or secondary diagnosis, 60,039 patients were assigned only G20 diagnosis code, whereas 36,835 had at least one G21-26 diagnosis as well in addition to the G20 code over this 10-year period. During these 10 years 62,108 patients were registered with any of the G21-26 diagnoses without any appearance with G20 code.

### Validating the Clinical Diagnosis Criteria of PD on a Smaller Subsample

For comparing inpatient and outpatient records in the hospital IT system (MedSol) and the large national database (NEUROHUN) we found the following (Tables 1, 2):

- out of 60 inpatients with G20 in any diagnosis position in the hospital records 59 appeared also in the national database. The one missing patient from the national database was due to a reporting error from the hospital.
- all of the 31 inpatients in the national database with G20 appearing at least in 2 years in the national database with one of these appearances in the period of Nov.–Dec. 2012 were present in the local hospital records.
- out of 224 outpatients with G20 in the hospital records 223 appeared in the national database. The one missing patient had no patient ID in the hospital records.
- all of the 224 outpatients with G20 appearing at least in 2 years in the national database with one of these appearances in the period of Nov.–Dec. 2012 were present in the hospital records.

**Table 1:** Number of inpatient record appearances in the hospital IT system (MedSol), the large national database (NEUROHUN) and vice-versa.

**Database**	**MedSol → NEUROHUN**	**NEUROHUN → MedSol**
Inpatient appearance	60 → 59	31 → 31
Correspondence	98.3%	100%

**Table 2:** Number of outpatient record appearances in the hospital IT system (MedSol), the large national database (NEUROHUN) and vice-versa.

**Database**	**MedSol → NEUROHUN**	**NEUROHUN → MedSol**
Outpatient appearance	224 → 223	224 → 224
Correspondence	99%	100%

When comparing medical documents to the national database, starting from 01 November 2012 until 31 December 2012 we examined final hospital discharge reports from MedSol of the 31 inpatients and consecutive 19 outpatients to reach total number of 50 patients, who appeared in NEUROHUN with 2-year G20 criteria. We found that out of 31 inpatients 26 (84%) had clinically supported PD. The other 5 patients had a final clinical diagnosis of as follows: multiple system atrophy (MSA) in one case, progressive supranuclear palsy (PSP) in 2 cases and in 2 cases other not defined secondary parkinsonian syndrome. Out of 19 outpatients 18 (94%) had clinically supported PD. One patient had other not defined secondary parkinsonian syndrome.

### Estimating the Number of Patients With Clinically Consistent PD Diagnosis for the Period of 2010–2012

Overall 46,383patients fulfilled our criteria for PD diagnosis between 2010 and 2012, i.e., they were registered in the neurological inpatient or outpatient system with G20 diagnosis at least twice and at least in 2 years of the study period. The diagnosis of PD was confirmed at least once by a neurology specialist in 43,009 cases (92%). Table 3 shows all the estimated crude and age standardized incidence and prevalence rates per year.

**Table 3:** Incidence (new patients per 100,000 inhabitants/year) and prevalence (number of patients per 100,000 inhabitants) of PD in Hungary between 2010 and 2012.

**Year**	**Crude incidence**	**Crude prevalence**	Standardized incidence^*^	Standardized prevalence^*^
2010	53	394	59	458
2011	50	406	57	473
2012	46	414	52	483
95% CI overall for 3 years	45–53	392–416	51–60	456–485

* *Age standardization was performed using the 2013 European standard population (17)*.

For the 3 years the mean crude incidence rate was 49/100,000/year (95% CI: 45–53), whereas the age standardized incidence was 56/100,000/year (95% CI: 51–60). Crude and age standardized mean prevalence rates were 404/100,000 (95% CI: 392–416) and 471/100,000 (95% CI: 456–485), respectively.

**Figure 1:**
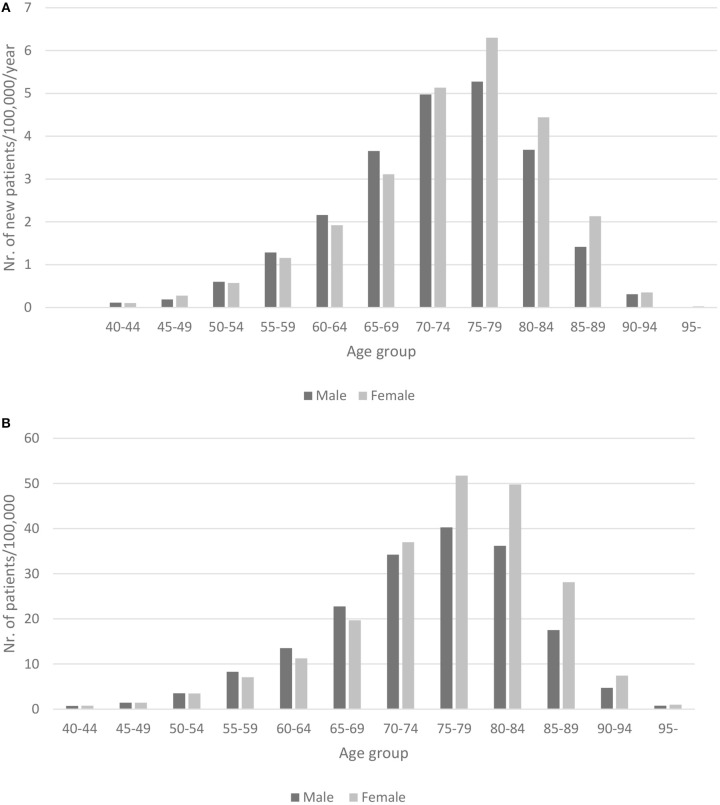
**(A)** Crude incidence of PD in Hungary between 2010 and 2012 (per 100,000/year) by 5 year age categories and gender. **(B)** Crude prevalence of PD in Hungary between 2010 and 2012 (per 100,000) by 5 year age categories and gender.

The crude rates of incidence and prevalence by 5-year age categories and by gender are shown in Figures 1A,B.

The male to female ratio of prevalence and incidence showed male dominance until 70 years of age after which female PD patients were more frequent.

### Linking the Clinically Consistent PD Cases With APD Medication Refill

**Figure 2:**
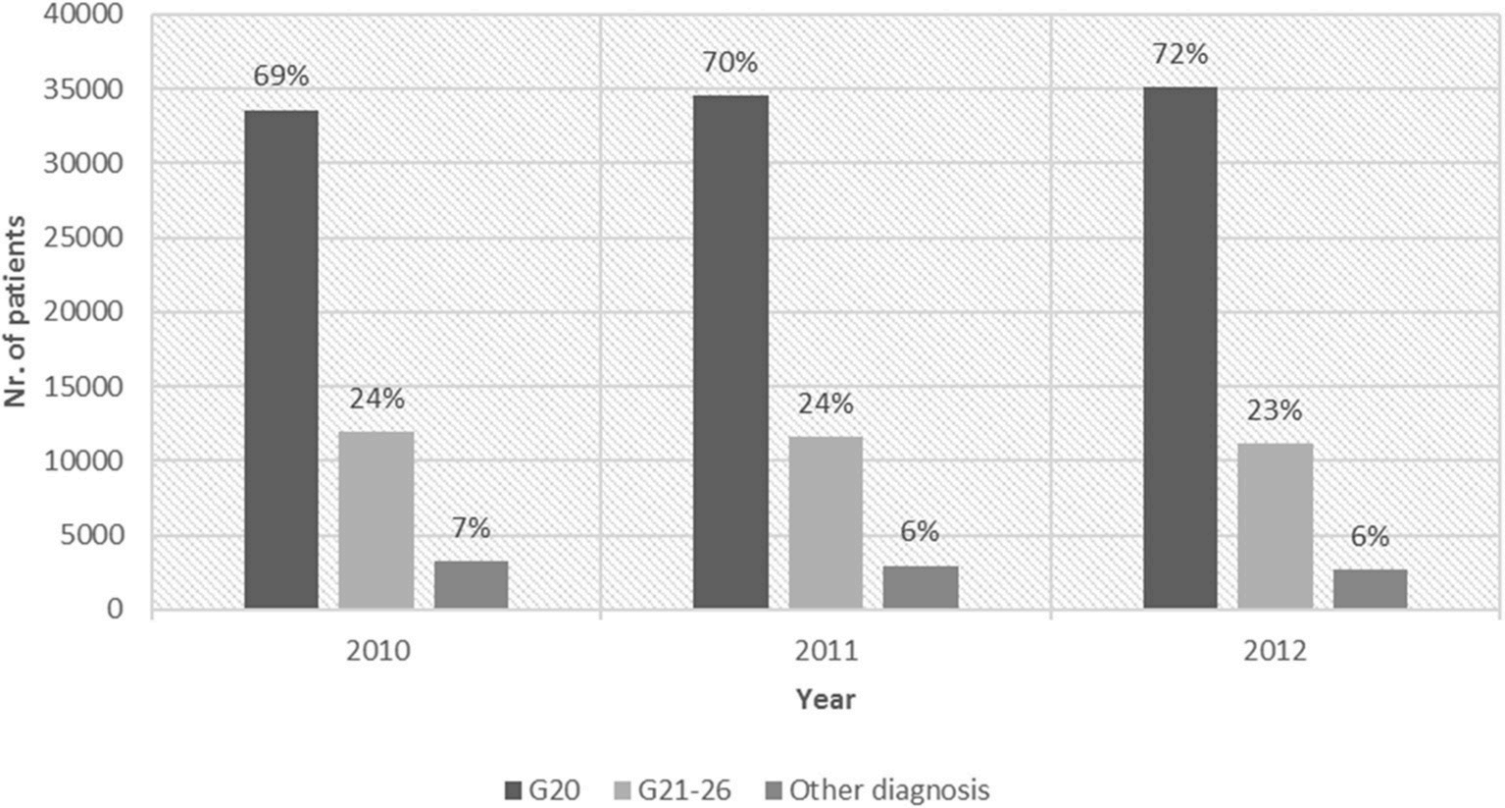
Refill of N04 ATC drugs: with G20 diagnosis, with G21–26 diagnosis and with other diagnosis codes between 2010 and 2012.

The number of patients who refilled annually N04 ATC prescriptions in the pharmacy database were 48,857, 49,113 and 49,041 in 2010, 2011, and 2012, respectively. The distribution of different diagnosis types for APD prescriptions each year are presented in Figure 2.

In the same 3 years the number of clinically consistent PD patients were 39,355, 40,608 and 41,382. The number of clinically consistent PD patients who also refilled APD medication was 28,541, 29,735, and 29,667 in these 3 years. The refill rate among those PD patients who also appeared at a neurology service in the same year was 0.80, whereas the refill rate of all living PD patients was 0.72.

## Discussion

In the present study our aim was to estimate the number of those with PD in Hungary and to validate prevalence and incidence rates by comparing two independent national healthcare databases. We found that incidence and prevalence estimates of PD are considerably higher than previous reports. A door-to-door survey in a district of Budapest performed 3 decades ago estimated an age standardized PD prevalence of 123.1 per 100,000 and age standardized incidence of 9.1 per 100,000 inhabitants per year (17). A European survey 20 years later gave higher numbers, suggesting 21.6 thousand PD patients in Hungary. We found even higher numbers, suggesting that in the years 2010–2012 in Hungary there are around 40,000 patients with PD. If we consider only those 72% who refill APD medication, the number is still considerably higher than previous estimates. This increase in incidence and prevalence values can be explained by the difference in the epidemiologic method used (more thorough and nationwide case identification) and by the growing frequency of PD recognition and patient admission to neurological services over the years.

The number of patients with physician-assigned PD diagnosis is confirmed by pharmacy refills of disease specific medications (N04 ATC) in overall 72% of the cases. The refill rate is higher (80%) among PD patients who receive regular neurological care. APDs in Hungary are used for PKM as well (ICD-10 diagnosis codes: G21–G26) in 23–24% of all the APD refills (Figure 2). Similarly to our findings, a report from Italy in 1998 (18) assumed that 75.2% of the total levodopa prescriptions were for patients with PD. A study with similar population-level case ascertainment methods like our design reported that the proportions of those with a levodopa prescription for PD diagnosis ranged from 39 to 66% (19). In Singapore 92.3% of PD patients were receiving levodopa (20). The 72% rate of APD refill in Hungary includes not only levodopa, but all N04 medications, like DOPA agonists as well.

A study from British Columbia, based on a prescription database with 97% coverage estimated crude PD prevalence rates ranging between 109 and 144 per 100,000 inhabitants (21). From the same region, a historical cohort study using administrative databases assessed the age-specific incidence of PD for persons 65 years or older, and found that the crude annual incidence rate was 252 per 100,000 person-years (22). A nationwide pharmacoepidemiological study conducted in Denmark using a drug tracer methodology from a national prescription database showed an age standardized prevalence rate for APD purchase of 164 persons per 100,000 and an incidence rate of 55 persons per 100,000 inhabitants/year concluding that prescriptions overestimated PD prevalence (23). In our study the majority of APDs were prescribed for PD, a quarter for PKM and a few cases for other diagnoses.

Estimating rates of PD in Israel, based on prescription database with 25% coverage of the population showed an incidence rate of 33 per 100,000/year and a prevalence rate which increased from 170 to 256 per 1000,000 over the years, indicating a burden of PD in Israel higher than previously assumed (24). We have also come to a similar conclusion.

In Hungary it was previously demonstrated that hospital discharge reports accurately identify patients with cerebrovascular diseases, if primary as well as secondary diagnoses are considered simultaneously during data analysis (25). As hospital reimbursement is based on the DRG system in Hungary, for some PD patients the primary diagnosis in the discharge report may be other than PD for financial considerations. However, PD also appears in the discharge report of these patients coded as a secondary diagnosis. Therefore, if hospital discharge reports are used to identify patients with PD, all discharge diagnosis categories, primary as well as secondary, should be considered.

Differentiating PD, especially at disease onset, from PKM is often difficult and challenging. Furthermore, PKMs are also frequently treated as PD due to responsiveness to dopaminergic therapy in some cases. Since society, patients, relatives and the health care system are similarly affected with PD and PKM, their merger may be justified from the disease burden perspective.

Although administrative data are a convenient and a relevant source for studying specific patient populations, it is a challenge to create a valid approach for correctly identifying patients with PD or PKMs. A report from 2010 found that the ICD-9 code for PKM has good sensitivity (75%) and excellent specificity (99.1%) (26), however a systematic review indicated that for PD/PKM the sensitivity of ICD codes was 18.7–100% and the specificity was between 0 and 99.9% (9). In a study about accuracy estimates, the authors concluded that administrative data were limited in the ability to identify PKM and distinguish between PD and PKM, however the addition of pharmacy data improved sensitivity. The authors also recommended identifying cases from multiple administrative databases and the use of algorithms to distinguish between the categories of PKM (13). Additionally, identifying a population of patients with PD from administrative databases represents a complicated issue for several other reasons which affect our study as well: (1) PD is a clinical diagnosis and it is definitively identified only by brain pathological findings (27), therefore the degree of clinical uncertainty always remains present. However, accuracy of the clinical diagnosis is likely to become more assured over time as clinical data accumulates therefore we used the 2 year diagnostic criteria; (2) PD diagnoses used in the study are not made by direct contact with the patients but by reliability on physicians/specialists reports prepared essentially for financial purposes; (3) As our results show, a greater number of persons are using N04 ATC medications than the estimated figure of persons with PD. The differences may partly be explained by the fact that N04 ATC drugs are not specific and unique medications for PD as they are also used for other conditions (e.g., drug-induced PKM, restless legs syndrome, tremor disorder, dopa-responsive dystonia - codes: G21–26) often less regularly and at lower doses; (4) the diagnosis is specified wrong on the prescription for a N04 ATC medication.

There are certain strengths of this study. First, we could analyze the database of a several years' time period with 100% coverage of the country with 10 million inhabitants, making this the largest Hungarian epidemiological study of PD. Second, while chronic diseases are often under-reported during hospital admissions where the condition may not be the primary cause of admission (28), the use of all categories of discharge diagnoses increases the efficacy of case identification. Third, by considering PD in all diagnostic categories of the discharge reports, the bias caused by financial considerations in reporting was diminished. Fourth, by cross-checking 2 independent databases—the clinical and the pharmacy repots—we could validate the accuracy of the diagnosis of PD.

This study also has several limitations. First, diagnostic accuracy may be limited by the lack of a direct individual clinical case certification by physical examination and individual chart review. Second, an overestimation of the number of PD patients may result from the application of PD diagnosis code (G20) or APD-based treatment (N04 ATC) for those who indeed had PKM. Third, underestimation of the number of those with PD may have occurred by exclusion of patients with clinical or pharmacy codes of PKM (G21–G26) who might indeed had PD. The possibility of misclassification error should be considered as an important source of research bias using health administrative databases and threatens the validity of study results (29). Additionally, patients not presenting in the neurological care system, undiagnosed patients, patients who have not yet been commenced on antiparkinsonian therapy, or those who do not refill the prescribed medication due to disadvantaged socioeconomic conditions result in further underestimation of the number of patients with PD.

In conclusion, the NEUROHUN database with proper case identification and case certification methodology can be appropriate to evaluate clinical, epidemiological, and organizational features of PD in Hungary. The higher incidence and prevalence values than previously estimated, reflect the growing burden of PD on the Hungarian health care system. The database has the potential to estimate quality of care, cost of care, follow-up and prevalence of PD or other specific neurological conditions in Hungary.

## Ethics Statement

All personal data protection regulations were followed, the Ethics Committee of Semmelweis University, Budapest, Hungary approved the study (Approval No: SE TUKEB 88/2015) and have therefore been performed in accordance with the ethical standards laid down in the 1964 Declaration of Helsinki and its later amendments.

## Author Contributions

SS, AA, and DB: study concept and design. AA, DB, and FO: acquisition of data. SS, MB, DB, FO, and AT: analysis and interpretation of data. SS and DB: drafting of the manuscript and approval of the final version. DB, SS, AA, MB, AT, and FO: critical revision of the manuscript for important intellectual content and approval of the final version.

### Conflict of Interest Statement

The authors declare that the research was conducted in the absence of any commercial or financial relationships that could be construed as a potential conflict of interest.
